# Glutathione-Responsive Folate-Targeted Prodrugs: Tumor-Specific PD-L1 and CD47 Blockade

**DOI:** 10.3390/molecules30214292

**Published:** 2025-11-05

**Authors:** Jianfeng Wang, Lianqi Liu, Dian Xiao, Fei Xie, Xinbo Zhou

**Affiliations:** Academy of Military Medical Sciences, Beijing 100850, China; ddwangjianfeng@163.com (J.W.); llianqi@126.com (L.L.)

**Keywords:** pro-drug, anti-PD-L1, anti-CD47, irAEs, glutathione responsiveness

## Abstract

Immune checkpoint inhibitors (ICIs) targeting PD-L1 and CD47 are clinically limited by severe off-target toxicities. To address this issue, immunotherapeutic prodrug strategies have been developed, aimed at preventing antibodies from binding to targets in healthy tissues and thereby reducing systemic toxicity. Existing strategies include prodrug technologies that mask the active sites of antibodies via peptide or polyethylene glycol (PEG) modification—yet these approaches also cause antibodies to lose their targeting ability. Herein, we propose an antibody prodrug strategy (termed FA-PEG-S-Ab) with active targeting capability. By modifying antibodies with folate-PEG-disulfide and PEG-disulfide linkages, we developed two novel prodrugs: FA-PEG-S-Atz (PD-L1-blocking prodrug) and FA-PEG-S-Hu5 (CD47-blocking prodrug). This strategy functions through two key steps: first, folate binding to folate receptor α (FRα)-mediated tumor-specific targeting enables the prodrugs to accumulate specifically in tumor tissues; subsequently, the high concentration of glutathione (GSH) in the tumor microenvironment (TME) specifically cleaves the disulfide bonds, removing the PEG shield, releasing the antibody, and restoring the antibody’s antigen-binding activity. In vitro experiments confirmed that the modified antibody prodrug FA-PEG-S-Hu5 exhibits high affinity for FRα (*K*_D_ = 4.02 × 10^−9^ M) and effectively masks the antibody’s binding activity (*K*_D_ from 1.05 × 10^−11^ M to 2.10 × 10^−8^ M). Following activation by GSH in the TME, this masking effect is reversed, and the antibody regains its binding affinity (*K*_D_ = 2.14 × 10^−10^ M). Crucially, FA-PEG-S-Hu5 completely eliminates hemolytic toxicity. This “folate targeting delivery + TME activation” prodrug strategy is expected to provide a new solution for addressing the off-target toxicities of conventional ICIs.

## 1. Introduction

Immune checkpoint inhibitors (ICIs) targeting PD-L1 and CD47 have become core modalities in cancer therapy. However, their clinical application remains constrained by off-target toxicity [1,2]. PD-L1 antibodies bind to physiologically expressed PD-L1 on normal tissues (e.g., lung epithelium, gastrointestinal mucosa), disrupting immune homeostasis and inducing immune-related adverse events (irAEs) in some patients [3].

CD47 antibodies (e.g., Hu5f9) face even more severe challenges: CD47 is ubiquitously expressed on the surface of red blood cells (RBCs), and the binding of CD47 antibodies to CD47 on RBCs causes hemolytic toxicity [4,5]. Magrolimab from Gilead, as the first CD47 antibody to enter clinical trials, exhibited effective antitumor activity against hematologic malignancies (e.g., acute myeloid leukemia, AML; higher-risk myelodysplastic syndrome, MDS) and solid tumors. However, subsequent phase III clinical studies were fully terminated due to failure to meet expected efficacy and increased risk of death [6,7]. Similarly, Evorpacept from ALX Oncology failed to demonstrate superior efficacy over chemotherapy in the ASPEN-02 clinical trial for MDS and was terminated due to safety concerns [8]. To date, no CD47 antibody has been approved for marketing, which highlights the challenges in the development of CD47 antibodies—namely, the severe hematologic toxicity caused by the widespread expression of CD47 on the surface of red blood cells and how to balance on-target efficacy and off-target risks [9].

To mitigate the off-target toxicity of immune checkpoint inhibitors (ICIs), a variety of strategies involving modification with peptides and polyethylene glycol (PEG) have been developed to date, but these strategies suffer from inherent drawbacks. For PD-L1 antibodies, tumor-penetrating peptides (TPPs, e.g., iRGD) enhance tumor accumulation by disrupting the stroma but do not alter the antibody’s binding affinity, leaving the antibody still able to bind normal tissues (e.g., lung epithelium) and trigger irAEs (e.g., pneumonitis) [10,11]. Conventional strategies for masking antibodies using polyethylene glycol (PEG) and peptides sterically hinder the antibodies’ antigen-binding fragments (Fab), resulting in decreased affinity [12,13]. While these methods effectively mask the antibody’s binding ability to the antigen, they also result in a loss of efficacy due to the lack of targeting, failing to address the issues associated with the application of immunosuppressive antibodies.

Folate receptor α (FRα) is a membrane protein that is highly expressed in various cancers (e.g., ovarian cancer, triple-negative breast cancer, lung cancer) but poorly expressed in normal tissues. This differential expression enables FRα-based tumor-targeted therapy to find applications in multiple fields [14,15,16,17,18,19]. For instance, FRα-targeted antibody-drug conjugates (ADCs) represented by mirvetuximab soravtansine have demonstrated favorable objective response rates in patients with platinum-resistant ovarian cancer in the MIRASOL clinical trial [20]. Additionally, FRα-targeted chimeras (FRTACs) as another FRα-targeted modality use folate molecules as targeting moieties to achieve precise targeting of FRα-positive tumors, thereby enabling efficient and selective degradation of membrane proteins in FRα-positive tumor cells and exhibiting favorable antitumor activity [21,22]. Alongside other FRα-targeted approaches, including liposomes and other nanocarriers as well as tumor diagnostic imaging, these strategies have become an important research direction in current precision oncology [23]. Theses outcomes validate the primary value of FRα in tumor-targeted therapy.

To further enhance the tumor specificity of immune checkpoint inhibitors (ICIs) and address off-target toxicity, we designed an antibody prodrug strategy based on folate targeting and GSH-responsive PEG shielding, termed FA-PEG-S-Ab. The core design of this strategy leverages two key attributes: the high expression of FRα on tumor cells, and the unique biochemical properties of the tumor microenvironment (TME). Compared with normal tissues, the TME has a significantly higher GSH concentration and an acidic pH, whereas the pH is neutral in normal tissues [24,25,26]. These features enable FA-PEG-S-Ab to achieve targeted delivery to tumor sites and selective activation [27,28,29,30].

**Scheme 1:**
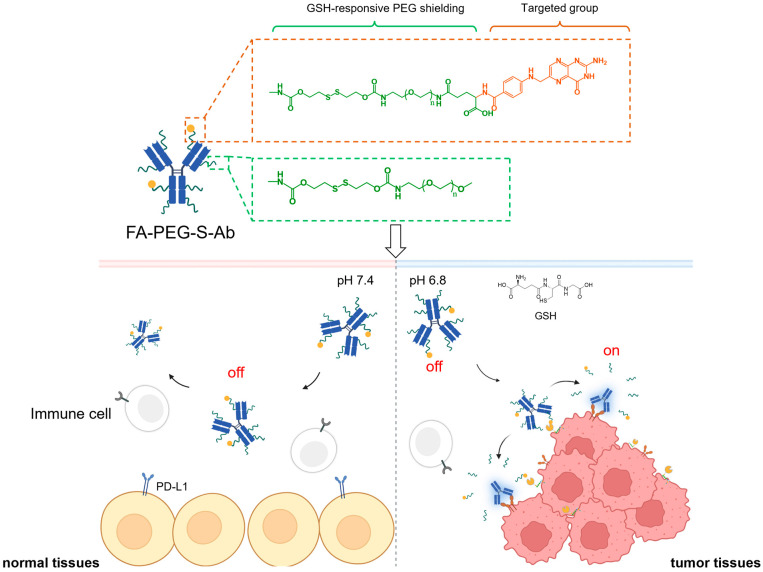
Schematic illustration of glutathione (GSH)-responsive, folate-targeted prodrugs (FA-PEG-S-Ab) enabling tumor-specific PD-L1/CD47 blockade. FA-PEG-S-Ab comprise two key modules: a folate-based targeted group (orange dashed box) for tumor-directed delivery via FRα binding, and a polyethylene glycol (PEG) shielding group (green dashed box) to mask antibody–antigen-binding sites. In normal tissues (pH 7.4, low GSH), the PEG shield remains intact, keeping prodrugs in an “off” state to avoid binding PD-L1/CD47 on normal/immune cells and prevent off-target toxicity. In tumor tissues (pH 6.8, high GSH), GSH cleaves disulfide linkers in both targeted and PEG moieties: this removes the PEG shield, dissociates the folate-targeted group, and activates prodrugs to an “on” state—allowing binding to PD-L1/CD47 on tumor cells for effective immune checkpoint blockade.

Using this strategy, we developed two prodrugs in this study: FA-PEG-S-Atz (an atezolizumab prodrug targeting PD-L1) and FA-PEG-S-Hu5 (a Hu5f9 prodrug targeting CD47). Once the prodrugs enter the circulatory system, they remain in an “off” state under normal physiological conditions (pH 7.4, low GSH): the PEG chains exert a shielding effect, reducing their binding to normal tissue cells. Meanwhile, the prodrugs can specifically target FRα-positive tumors through folate-FRα interactions. Due to the acidic nature of the TME (pH 6.8) and its high GSH levels, the disulfide bond is cleaved, the PEG long chain dissociates from the antibody and the antibody function is subsequently restored (Scheme 1). This dual-regulation mechanism, which first achieves tumor-specific targeting and then enables microenvironment-dependent activation, is expected to overcome the limitations of traditional strategies, ensuring that the prodrug exhibits excellent targeting and the antibody function is restored only at the intended site of action.

## 2. Results

### 2.1. Design and Synthesis of FA-PEG-S-Ab

**Figure 1:**
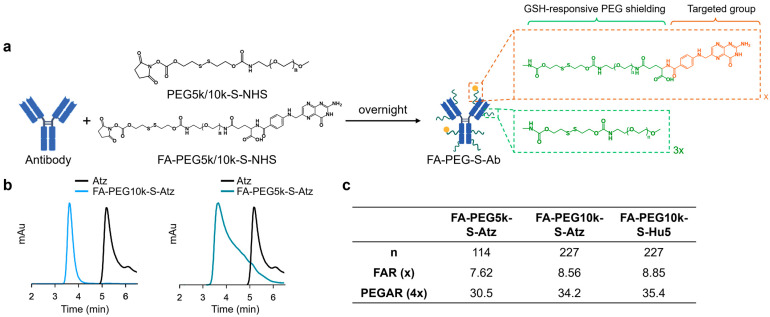
Synthesis and characterization of FA-PEG-S-Ab. (**a**) The synthesis of FA-PEG-S-Ab. (**b**) Size-exclusion chromatography (SEC) profiles of Atz, FA-PEG5k-S-Atz, and FA-PEG10k-S-Atz showing peak shifts. (**c**) The folate-PEG-to-antibody ratio (FAR) and PEG shielding-to-antibody ratio (PEGAR) of FA-PEG-S-Ab.

FA-PEG-S-Ab were constructed by conjugating folate moieties and PEG shielding to native antibodies (Atz or Hu5) via GSH-cleavable linkers (Figure 1a,b). Natural antibodies typically contain over 80 lysine residues available for conjugation [31]. Thus, to achieve the optimal shielding effect on the antibodies, we utilized amino conjugation, which is achieved by reacting Atz or Hu5 with a mixture of GSH-responsive folate-PEG5k/10k-NHS and PEG5k/10k-NHS at a molar ratio of 25:75 (total molar ratio 100:1) (Figures S1 and S2, and Table S1), controlling the feed ratio to achieve the desired modification levels. The conjugation reaction was monitored by size-exclusion chromatography (SEC) to confirm reaction completion.

The number of conjugated folate and PEG moieties was determined as follows: the folate-PEG-to-antibody ratio (FAR) was quantified by ultraviolet (UV) spectroscopy, utilizing characteristic absorbances at 370 nm (folate) and 280 nm (antibody) to calculate the FAR. Based on the 25:75 molar ratio of folate-PEG-NHS to PEG-NHS in the reaction feed and assuming comparable reactivity of the two NHS esters toward antibody amino groups, the PEG shielding-to-antibody ratio (PEGAR) was derived as PEGAR = FAR × 4. These analyses confirmed that both FA-PEG5k/10k-S-Atz and FA-PEG10k-S-Hu5 contained approximately 8 folate moieties and 32 PEG chains per antibody, with nearly half of the lysine residues on each antibody successfully modified (Figure 1c).

### 2.2. Structure-Activity Relationship (SAR) of FA-PEG-S-Ab

**Figure 2:**
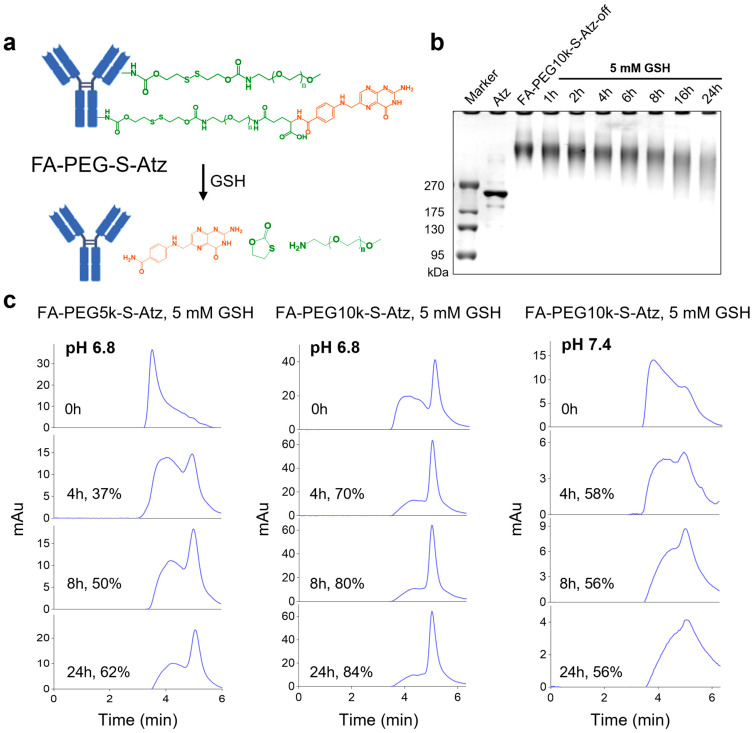
GSH-responsive activation and characterization of FA-PEG-S-Atz. (**a**) Schematic illustration of GSH-mediated cleavage of PEG shielding chains and folate-targeted group in FA-PEG-S-Atz, restoring the antibody structure. (**b**) Sodium dodecyl sulfate-polyacrylamide gel electrophoresis (SDS-PAGE) analysis of FA-PEG10k-S-Atz-off incubated with 5 mM GSH over time, demonstrating time-dependent PEG detachment. (**c**) HPLC analysis of FA-PEG5k-S-Atz and FA-PEG10k-S-Atz incubated with 10 mM GSH at pH 6.8 and pH 7.4, quantifying the impact of PEG chain length and pH on GSH-mediated cleavage efficiency.

To further analyze the effect of PEG chain length on the shielding efficiency of antibody binding sites in FA-PEG-S-Ab, we took FA-PEG-S-Atz as a model and synthesized FA-PEG5k-S-Atz and FA-PEG10k-S-Atz with different PEG chain lengths. Subsequently, we used SDS-PAGE to monitor whether FA-PEG-S-Atz could smoothly switch from the “off” state to the “on” state upon GSH treatment. The results showed that when FA-PEG10k-S-Atz was incubated with 5 mM GSH, its band gradually migrated to the molecular weight region of Atz over time. This confirmed that GSH could successfully induce the cleavage of disulfide bonds and the release of PEG chains, with the detachment of PEG chains being time-dependent (Figure 2a,b).

To investigate the effects of PEG chain length and pH on cleavage efficiency, we treated FA-PEG5k-S-Atz and FA-PEG10k-S-Atz with 5 mM GSH under conditions mimicking the in vivo environment (pH 6.8 for tumor-mimetic and pH 7.4 for blood-mimetic) and analyzed their cleavage efficiencies. At pH 6.8, FA-PEG10k-S-Atz showed higher cleavage efficiency than FA-PEG5k-S-Atz; for instance, the cleavage rates at 24 h were approximately 84% and 62%, respectively. In contrast, at pH 7.4, the cleavage efficiency of FA-PEG10k-S-Atz decreased significantly, reaching only about 56% at 24 h (Figure 2c). These results indicate that longer PEG chains and the acidic tumor microenvironment synergistically promote GSH-mediated PEG detachment.

**Figure 3:**
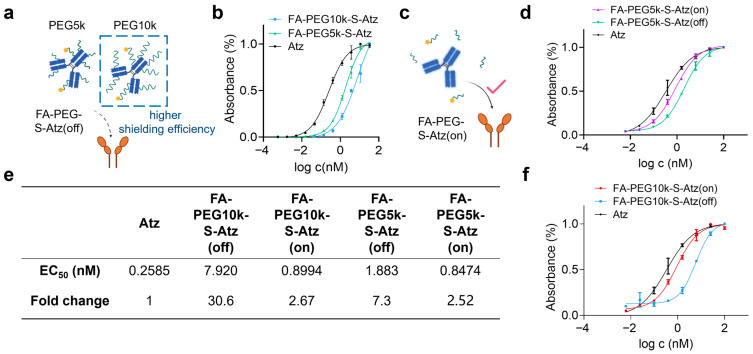
Binding activity of FA-PEG-S-Atz regulated by GSH. (**a**) Schematic showing impaired PD-L1 binding of FA-PEG-S-Atz-off (intact PEG shielding). (**b**) ELISA binding curves of Atz, FA-PEG5k-S-Atz, and FA-PEG10k-S-Atz to PD-L1. (**c**) Schematic of restored PD-L1 binding in the “on” state after GSH-mediated PEG cleavage. (**d**,**f**) ELISA binding curves of FA-PEG5k-S-Atz (on), FA-PEG5k-S-Atz (off), FA-PEG10k-S-Atz (on), FA-PEG10k-S-Atz (off), and Atz to PD-L1, showing recovered binding after GSH treatment. (**e**) Summary of EC_50_ values and fold changes from (**b**,**d**,**f**), quantifying binding affinity restoration.

Furthermore, we used enzyme-linked immunosorbent assay (ELISA) to investigate the effect of PEG chain length on antibody binding activity. FA-PEG5k/10k-S-Atz in the “off” state showed significantly reduced binding activity to PD-L1 compared with unmodified Atz, and FA-PEG10k-S-Atz exhibited the most significant change in binding activity (Figure 3a,b). After treating FA-PEG5k/10k-S-Atz with 5 mM GSH to cleave the PEG chains, ELISA results showed that the binding activity was restored (Figure 3c). FA-PEG5k/10k-S-Atz in the “on” state exhibited binding curves similarly to that of Atz (Figure 3d,f). Quantitative analysis further confirmed these findings. In the off state, the half-maximal effective concentration (EC_50_) values of FA-PEG5k-S-Atz and FA-PEG10k-S-Atz were 1.883 nM and 7.920 nM, respectively, which were approximately 7.3-fold and 30.6-fold higher than that of Atz. In the on state, the EC_50_ values of FA-PEG5k-S-Atz and FA-PEG10k-S-Atz were 0.8474 nM and 0.8944 nM, respectively, increasing by approximately 2.52-fold and 2.67-fold compared with Atz (Figure 3e). These results demonstrate that PEG10k exhibits stronger shielding performance than PEG5k, indicating that PEG chain length is an effective and tunable parameter for reducing off-target binding and balancing the shielding efficiency of prodrugs. Consequently, PEG10k was selected as the preferred choice for subsequent studies on CD47-targeted prodrugs.

### 2.3. Characterization of FA-PEG10k-S-Hu5

**Figure 4:**
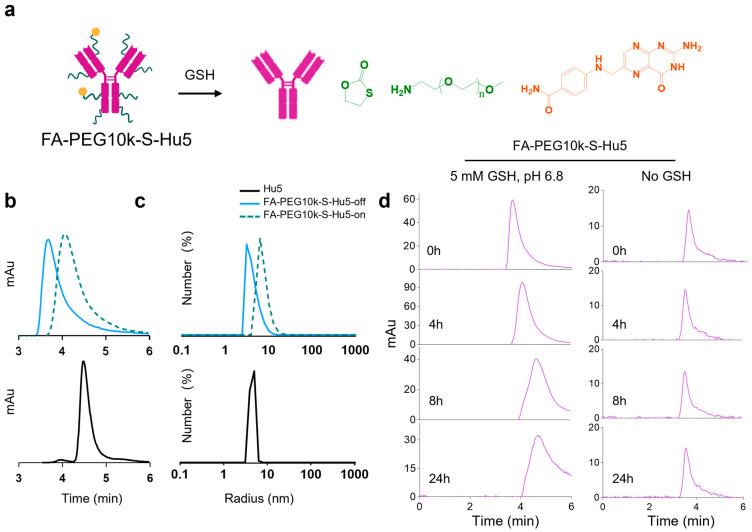
GSH-responsive characterization of FA-PEG10k-S-Hu5. (**a**) Schematic illustration of FA-PEG10k-S-Hu5 with GSH-cleavable targeting group and PEG shielding chain, undergoing disulfide bond cleavage upon GSH exposure. (**b**) HPLC profiles of Hu5, FA-PEG10k-S-Hu5-off (without GSH), and FA-PEG10k-S-Hu5-on (with 5 mM GSH), confirming conjugation and GSH-induced cleavage (peak shift toward Hu5). (**c**) Dynamic light scattering (DLS) profiles of Hu5, FA-PEG10k-S-Hu5 (off), and FA-PEG10k-S-Hu5 (on), showing changes in hydrodynamic radius upon GSH treatment. (**d**) Time-course HPLC analysis of FA-PEG10k-S-Hu5 incubated with 5 mM GSH at pH 6.8 (**left panel**) and without GSH (**right panel**) over 0, 4, 8, and 24 h, verifying time-dependent GSH-responsive cleavage and stability in the absence of no GSH.

Based on the study of PEG chain length in FA-PEG-S-Atz, we synthesized the PEG10k-based FA-PEG10k-S-Hu5. Size exclusion chromatography (SEC) results showed significant differences in elution times between Hu5, FA-PEG10k-S-Hu5-off, and FA-PEG10k-S-Hu5-on, confirming the successful conjugation of PEG and the GSH-induced cleavage of disulfide bonds (Figure 4a,b). Further dynamic light scattering (DLS) analysis indicated that the hydrodynamic radius of FA-PEG10k-S-Hu5-off was larger than that of Hu5. In contrast, FA-PEG10k-S-Hu5-on (after GSH treatment) exhibited a particle size distribution similarly to that of Hu5, which suggested that the disulfide bonds had cleaved and the long-chain PEG had dissociated (Figure 4c).

Additionally, under pH 6.8 conditions mimicking the tumor microenvironment, analyses were performed on FA-PEG10k-S-Hu5 incubated with 5 mM GSH. The results revealed that the cleavage process of FA-PEG10k-S-Hu5 was time-dependent: its characteristic peak gradually diminished and its elution time was delayed over time. In contrast, under neutral conditions without GSH, the retention time of FA-PEG10k-S-Hu5’s characteristic peak remained unchanged (Figure 4d). These results confirm that FA-PEG10k-S-Hu5 undergoes GSH-mediated, time-dependent structural activation in the tumor microenvironment.

### 2.4. GSH Responsiveness of FA-PEG10k-S-Hu5

**Figure 5:**
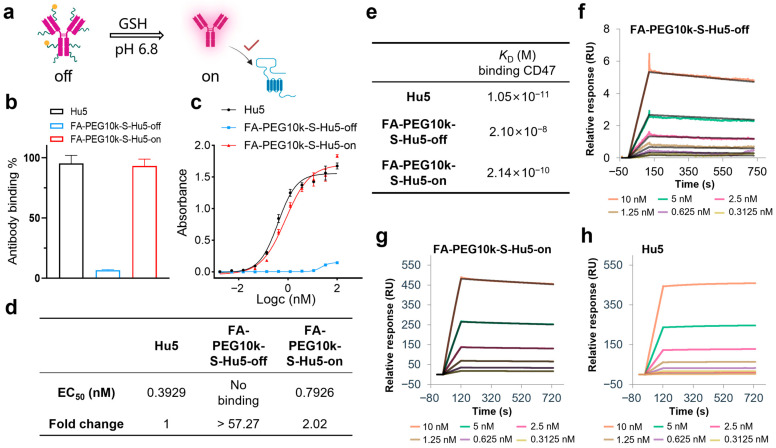
Binding activity of FA-PEG10k-S-Hu5 regulated by GSH. (**a**) Schematic showing “off” state and GSH-activated “on” state of FA-PEG10k-S-Hu5 for target binding at pH 6.8. (**b**) ELISA binding assay of Hu5, FA-PEG10k-S-Hu5-off, and FA-PEG10k-S-Hu5-on (33.33 nM) to CD47, indicating restored binding after GSH treatment. (**c**) Dose-dependent ELISA binding curves of Hu5, FA-PEG10k-S-Hu5-off, and FA-PEG10k-S-Hu5-on to CD47. (**d**) Summary of EC_50_ values and fold changes from (**c**), quantifying binding affinity restoration. (**e**) Summary of binding affinities (*K*_D_) for Hu5, FA-PEG10k-S-Hu5-off, FA-PEG10k-S-Hu5-on, and folate to CD47, derived from (**f**–**h**). (**f**–**h**) Surface plasmon resonance (SPR) analysis of FA-PEG10k-S-Hu5-off (**f**), FA-PEG10k-S-Hu5-on (**g**), and Hu5 (**h**) binding to CD47.

In non-tumor environments, FA-PEG10k-S-Hu5 exists at off state. However, under pH 6.5 conditions mimicking the tumor microenvironment, it switches to the on state upon exposure to GSH and restores its binding ability (Figure 5a). ELISA results showed that at a concentration of 33.33 nM, FA-PEG10k-S-Hu5-off exhibited minimal binding ability to CD47, while FA-PEG10k-S-Hu5-on (after GSH treatment) bound to CD47 at a level comparable to that of Hu5 (Figure 5b). Dose-dependent ELISA curves and quantitative analysis further confirmed this observation: FA-PEG10k-S-Hu5-off showed almost undetectable CD47 binding activity, with an EC_50_ value more than 57.27-fold higher than that of Hu5. In contrast, FA-PEG10k-S-Hu5 in the on state recovered its binding ability, and its EC_50_ value (0.7928 nM) was only approximately 2.02-fold higher than that of Hu5 (0.3929 nM) (Figure 5c,d). To further clarify the changes in antibody affinity before and after GSH regulation, we performed surface plasmon resonance (SPR) assays (Figure 5e). The affinity of FA-PEG10k-S-Hu5 in the off state for CD47 was significantly reduced due to PEG shielding (*K*_D_ = 2.10 × 10^−8^ M) (Figure 5f). After cleavage with 5 mM GSH, the affinity of FA-PEG10k-S-Hu5 in the on state for CD47 was restored to a level close to that of Hu5 (*K*_D_ = 2.14 × 10^−10^ M) (Figure 5g). Notably, Hu5 itself exhibited extremely high affinity for CD47 (*K*_D_ = 1.05 × 10^−11^ M) (Figure 5h).

These results clearly illustrate the mechanism of FA-PEG10k-S-Hu5: in the off state, while PEG shielding inhibits CD47 binding; in the on state, GSH triggers the dissociation of PEG and folate, thereby restoring CD47-binding ability.

### 2.5. Folate-Mediated Targeted Cellular Binding of FA-PEG10k-S-Hu5

**Figure 6:**
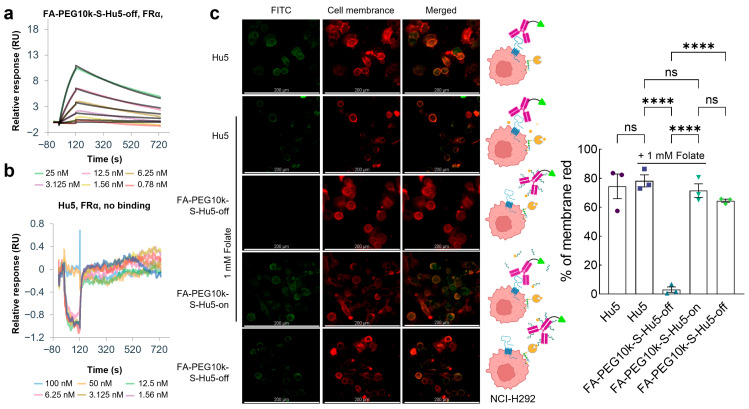
Folate targeting and cellular binding assay of FA-PEG10k-S-Hu5. (**a**,**b**) Surface plasmon resonance (SPR) analysis of FA-PEG10k-S-Hu5-off and Hu5 binding to FRα. (**c**) Fluorescence microscopy images of NCI-H292 cells treated with Hu5 or FA-PEG10k-S-Hu5-off under conditions with or without folate competition, while those treated with FA-PEG10k-S-Hu5-on served as the control. Columns show FITC fluorescence, cell membrane staining (red), and merged images; schematics on the middle illustrate binding mechanisms for each group, Scale bar: 200 μm. The percentage of membrane red from each group was quantitated using ImageJ 1.53t, and the data are presented as mean ± SEM of three independent experiments. *p* values were calculated using a two-tailed Student’s *t* test (**c**). ns, not significant. **** *p* < 0.0001.

To verify the folate-mediated targeted cellular binding property of FA-PEG10k-S-Hu5, we first evaluated its binding ability to FRα. Results showed that FA-PEG10k-S-Hu5 in the off state acquired FRα-binding capacity, which was directly mediated by folate (*K*_D_ = 4.02 × 10^−9^ M) (Figure 6a). In sharp contrast, Hu5 exhibited no binding activity to FRα at all (Figure 6b). These findings clearly confirm that the FRα-binding ability of FA-PEG10k-S-Hu5 is entirely dependent on folate modification, laying the foundation for its folate-mediated targeted binding to tumor cells.

We further investigated the cellular binding pattern of FA-PEG10k-S-Hu5 through FITC labeling and co-incubation with NCI-H292 cells under different conditions (Figure 6c). For comparison, Hu5 and Hu5 (with 1 mM folate) showed strong membrane-localized fluorescence, which was attributed to its direct binding to CD47 on tumor cell surfaces but had no correlation with folate-mediated targeting. In contrast, when FA-PEG10k-S-Hu5-off was co-incubated with excess free folate, no FITC fluorescence was detected on NCI-H292 cells. This result was due to dual effects: PEG shielding inhibited CD47 binding, while free folate competed with FA-PEG10k-S-Hu5 for FRα, ultimately abolishing its folate-mediated targeted binding to cells. After GSH treatment, the PEG shielding layer dissociated, allowing FA-PEG10k-S-Hu5-on to simultaneously bind to FRα and CD47, exhibiting the strongest membrane fluorescence. Notably, when FA-PEG10k-S-Hu5-off was incubated without free folate competition, but detectable membrane fluorescence was observed. This indicated that even with PEG-mediated CD47 binding inhibition, FA-PEG10k-S-Hu5 could still achieve targeted binding to NCI-H292 cells through folate-FRα interaction.

These results demonstrate that FA-PEG10k-S-Hu5 has the potential to achieve targeted accumulation in tumor tissues via folate-FRα binding by leveraging the differential expression of FR between tumor and normal tissues, and upon exposure to the tumor microenvironment, the shielding is removed to restore the antibody’s CD47-binding activity.

### 2.6. Hemolysis Safety of FA-PEG10k-S-Hu5

**Figure 7:**
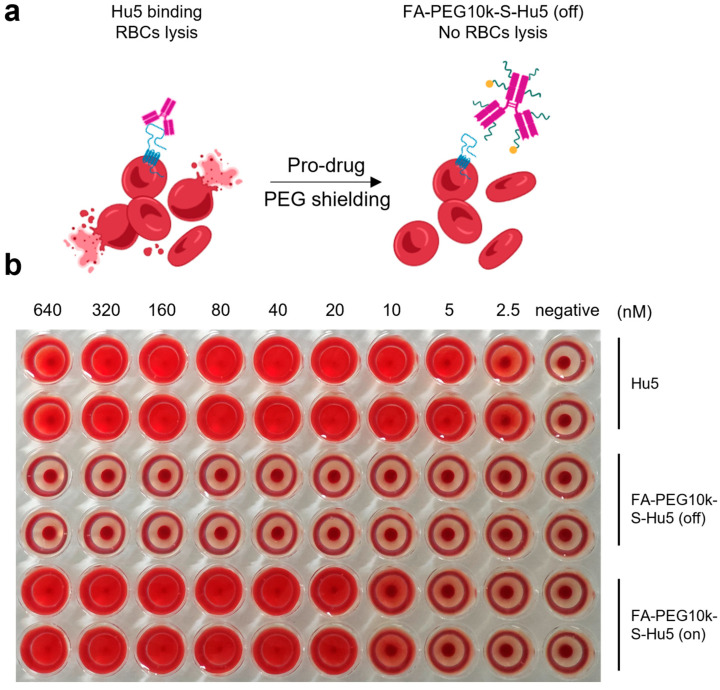
The hemolysis assay of FA-PEG10k-S-Hu5. (**a**) Schematic of hemolysis assay: Hu5 binds CD47 on red blood cells (RBCs) to induce lysis, while FA-PEG10k-S-Hu5 (off) (PEG-shielded) does not induce lysis. (**b**) Red blood cells (RBCs) were incubated with serial dilutions of Hu5 (top two rows), FA-PEG10k-S-Hu5 (off) (middle two rows), and FA-PEG10k-S-Hu5 (on) (bottom two rows) at concentrations ranging from 2.5 to 640 nM (with PBS as a negative control).

To further confirm the safety of FA-PEG10k-S-Hu5, we conducted a hemolysis assay, in which FA-PEG10k-S-Hu5 and Hu5 were separately co-cultured with red blood cells (RBCs) at gradient concentrations (Figure 7a). As expected, Hu5 induced dose-dependent hemolysis, as indicated by the gradual increase in the severity of RBCs hemolysis with rising Hu5 concentration. In contrast, FA-PEG10k-S-Hu5 showed no hemolysis at any tested concentration, consistent with the negative control group—even at the highest concentration (640 nM) (Figure 7b). This is attributed to the shielding effect of FA-PEG10k-S-Hu5, which reduces its binding to RBCs. Notably, after treatment with 5 mM glutathione (GSH) for 24 h, the side chains of FA-PEG10k-S-Hu5 cleaved and dissociated, switching the compound from the “off” to the “on” state. This restored the binding affinity of the Hu5 antibody to RBCs, resulting in hemolytic toxicity. These results confirm that FA-PEG10k-S-Hu5, designed based on our prodrug strategy, can effectively alleviate the off-target toxicity of the Hu5 monoclonal antibody toward RBCs, thereby enhancing its safety.

## 3. Discussion

This study proposes a novel prodrug strategy termed FA-PEG-S-Ab, which integrates folate targeting and GSH-responsive polyethylene glycol (PEG) shielding. We selected atezolizumab (anti-PD-L1) and Hu5F9 (anti-CD47), which previous studies have reported to exhibit potent in vivo antitumor activity through target blockade, to verify the effectiveness of our strategy.

To implement this strategy, we constructed FA-PEG-S-Ab, including FA-PEG-S-Atz and FA-PEG-S-Hu5. Its core structure consists of four covalently linked modules: the antibody as the functional core, folate moieties for FRα targeting, tunable PEG chains (PEG5k/PEG10k) for shielding, and GSH-cleavable disulfide linkers that connect all components. This design ensures that folate mediates FRα-targeted delivery, while PEG shields the antibody’s antigen-binding regions through steric hindrance. In contrast, the GSH-cleavable linkers can specifically remove the shielding in the tumor microenvironment (TME), thereby restoring antibody function.

In in vitro experiments, the folate moieties enabled FA-PEG-S-Ab to bind to tumor cells in a targeted manner. The PEG chains effectively shielded the antibody’s binding activity, achieving a maximum reduction of over 57-fold and a three-order-of-magnitude decrease in affinity. After GSH treatment, the disulfide linkers were efficiently cleaved, releasing the PEG shielding chains and restoring the antibody’s binding activity, where the binding activity differed by no more than 3-fold before and after restoration. The PEG shielding modification strategy precisely attenuates rather than eliminates the Hu5 binding to CD47. This controllable regulation has successfully abrogated hemolytic toxicity while preserving the ability for binding functional recovery under tumor-specific reductive conditions.

This study has inherent limitations, primarily the focus on in vitro validation. Future work will be extended to in vivo studies to evaluate tumor accumulation and therapeutic efficacy, such as using CD47-humanized mouse models bearing stably CD47-transfected RM-1 prostate cancer tumors (FRα+/PD-L1+). The combination of FA-PEG-S-Atz and FA-PEG-S-Hu5 merits exploration: FA-PEG-S-Atz reinvigorates T-cell immunity by blocking PD-L1, while FA-PEG-S-Hu5 activates macrophage-mediated phagocytosis by blocking CD47; their synergistic effect may reshape the immunosuppressive TME and achieve superior antitumor efficacy.

## 4. Materials and Methods

### 4.1. Synthesis of FA-PEG-S-Ab

For the synthesis of FA-PEG-S-Ab, 5.0 mg/mL solutions of antibody (Atz and Hu5) were each reacted with a total of 100 equivalents of functionalized NHS esters in PBS (pH 7.4). The NHS ester mixtures consisted of 75% GSH-responsive PEG-NHS (4.5 mg/mL in PBS) and 25% targeting moiety-NHS for both conjugates: specifically, folate-PEG5/10k-NHS (prepared in dimethyl sulfoxide at 5 mg/mL) for FA-PEG-S-Ab, and a GSH-cleavable targeting group-NHS (prepared in dimethyl sulfoxide at 5 mg/mL) for FA-PEG10k-S-Hu5. Reaction mixtures were incubated overnight at ambient temperature under gentle stirring, then purified via ultrafiltration (5500 rpm, 15 min per cycle, repeated three times) using Amicon centrifugal filters (molecular weight cutoff, 50 kDa) with PBS (pH 7.4) as the exchange buffer to remove unreacted reagents. The resulting FA-PEG-S-Ab were collected and stored at 4 °C until use.

### 4.2. UV-Vis Spectroscopy

To determine the number of folate and PEG moieties conjugated to FA-PEG-S-Ab, the folate-PEG-to-antibody ratio (FAR) and PEG-to-antibody ratio (PEGAR) were calculated as follows [21]:

For folate conjugation quantification, UV-visible spectroscopy was used. Folate-PEG10000-NHS, native antibodies (Atz or Hu5), and prodrugs (FA-PEG-S-Ab) were diluted in filtered PBS (pH 7.4). Absorption spectra were scanned over 190–400 nm, and molar extinction coefficients were determined at 280 nm (characteristic of protein) and 370 nm (characteristic of folate). The FAR was calculated using these extinction coefficients with the established formula.

For PEG conjugation quantification, the PEG-to-antibody ratio (PEGAR) was derived based on the feed ratio of PEG-NHS to folate-NHS during synthesis (75:25, i.e., 3:1 molar ratio). Assuming comparable reactivity of the two NHS esters toward antibody amino groups, the PEGAR was calculated as PEGAR = FAR × 4, where FAR represents the experimentally determined folate-PEG-to-antibody ratio. This approach leverages the stoichiometric relationship of the starting materials to derive the PEG conjugation level.

A_370nm_ = ε_folate-PEG5k/10k-NHS, 370nm_ × C_folate-PEG5k/10k-NHS_ (1)

A_280nm_ = ε_folate-PEG5k/10k-NHS, 280nm_ × C_folate-PEG5k/10k-NHS_ + ε _mAb, 280nm_ × C_mAb_ (2)

FAR = C_folate-PEG5k/10k-NHS_/C_mAb_ (3)

ε_folate-PEG5k/10k-NHS_, 370 nm, molar extinction coefficient for folate-PEG5k/10k-NHS at 370 nm.

ε_folate-PEG5k/10k-NHS_, 280 nm, molar extinction coefficient for folate-PEG5k/10k-NHS at 280 nm.

ε_mAb_, 280 nm, molar extinction coefficient for antibody at 280nm.

A_370nm_, absorption intensity for prodrugs at 370 nm.

A_280nm_, absorption intensity for prodrugs at 280 nm.

C_folate-PEG5k/10k-NHS_, concentration of folate-PEG10000-NHS on the prodrugs.

C_mAb_, concentration of antibody on the prodrugs.

### 4.3. Size-Exclusion Chromatography (SEC)

FA-PEG-S-Ab and native antibodies (Atz, Hu5) were diluted to 1 mg/mL in PBS (pH 7.4). For samples subjected to GSH treatment, they were incubated with 5 mM GSH at 37 °C for 1 h prior to dilution. Separation was performed using an Agilent 1260 HPLC system (Agilent Technologies, Santa Clara, CA, USA) equipped with a TSKgel UP-SW3000 column (4.6 mm × 10 cm, 2.5 μm; Tosoh, Corporation, Nanyo, Japan). The mobile phase contained 150 mM NaCl, and the flow rate was maintained at 0.2 mL/min. The elution process was monitored by UV absorption at 280 nm (for protein detection) using a diode array detector. For time-course cleavage assays (e.g., FA-PEG-S-Ab incubated with GSH at pH 6.8), samples were collected at designated time points, diluted to 1 mg/mL in PBS (pH 7.4), and analyzed with the same HPLC configuration to monitor alterations in elution profiles.

### 4.4. ELISA Assays

An enzyme-linked immunosorbent assay (ELISA) was performed to evaluate the binding ability of antibodies to their target proteins. Briefly, 96-well plates were coated with recombinant target proteins (1 µg/mL in PBS) overnight at 4 °C, followed by blocking with 5% non-fat milk in PBS for 1 h at room temperature. Serial dilutions of antibodies were added and incubated for 2 h, and bound antibodies were detected with HRP-conjugated secondary antibodies and TMB substrate. The absorbance was measured at an optical density (OD) of 450 nm using a microplate reader (Thermo Fisher Scientific, Inc., Waltham, MA, USA). Binding affinity was analyzed by plotting dose–response curves and calculating EC_50_ values with non-linear regression (GraphPad Prism 9.5.1).

### 4.5. SPR Assay

Binding affinities of the samples to their target proteins were measured using a Biacore 8K instrument (Cytiva, Washington, MA, USA) based on surface plasmon resonance (SPR). Target proteins were immobilized onto a CM5 sensor chip via standard amine coupling at 25 °C, with an immobilization buffer (PBS-T, 0.05% Tween-20) used throughout the process. The reference channel was activated and blocked to eliminate non-specific binding of compounds to the chip surface. Serial dilutions of samples were injected into the channels to assess binding affinity to the target proteins. Equilibrium dissociation constants (*K*_D_) were calculated by fitting the binding data to a 1:1 Langmuir binding model using Biacore 8K Evaluation Software (5.0.18.22102).

### 4.6. Particle Size Analysis

The size distribution of FA-PEG10k-S-Hu5 was determined by dynamic light scattering (DLS) using a Wyatt Technology NanoStar II instrument (DLS, WYATT, Santa Barbara, CA, USA).

### 4.7. Colocalization Live-Cell Imaging of FA-PEG10k-S-Hu5 by Fluorescence Microscopy

NCI-H292 cells were plated at a density of 10,000 cells per well on 8-well chamber slides 1 day prior to the experiment. For sample preparation: 30 nM FITC-labeled drugs, including Hu5 (control), FA-PEG10k-S-Hu5 -off, FA-PEG10k-S-Hu5 -on (pretreated with 5 mM GSH at 37 °C for 24 h to induce cleavage), and FA-PEG10k-S-Hu5 -off with excess free folate (1 mM, for competition assay) were prepared separately. Cells were incubated with each sample for 1 h at 37 °C, then washed 3 times with PBS to remove unbound proteins. To visualize cell membranes, cells were incubated with DiD (red fluorescent membrane dye) for 15 min at 37 °C. After three additional washes with PBS, live cells were imaged using a Leica DMi8 fluorescence microscope (Leica, Wetzlar, Germany). Fluorescence was excited using 488 nm (FITC) and 665 nm (DiD) lasers. Image analysis was performed using Leica view (3.7.9.20979) software and ImageJ 1.53t software to assess fluorescence colocalization and membrane association.

### 4.8. Hemolysis Assay

In the hemolysis assay without the involvement of macrophages, Hu5f9 (Hu5) may induce hemolysis through the following mechanisms: on the one hand, due to its high-affinity bivalent binding structure, it can bind to two CD47 proteins simultaneously, forming a Y-shaped conformation that triggers red blood cell agglutination and subsequent hemolysis; on the other hand, as an IgG4 antibody, Hu5 exhibits weak complement activity owing to its unique Fc region, yet it may still pose a certain hemolytic risk in practical applications [6,32,33,34,35].

Hu5, FA-PEG10k-S-Hu5 (off), and FA-PEG10k-S-Hu5 (on, pretreated with 5 mM GSH at 37 °C for 24 h to induce cleavage) were subjected to a twofold serial dilution with PBS buffer in a 96-well hemagglutination plate, generating concentrations ranging from 640 nM to 2.5 nM (with PBS as a negative control). Separately, human whole blood was mixed with Alsever’s solution (Solarbio, Cat R1016, Beijing, China) at a 1:1 ratio, washed twice with PBS, and then prepared into a 3% human erythrocyte suspension. Then, the 3% human erythrocyte suspension (50 μL) in PBS was added to each well containing the serially diluted samples. The plates were incubated at 37 °C for 2h. The extent of hemolysis was observed by assessing the red color intensity of the supernatant (indicating RBC lysis), and images of the plates were captured for documentation.

## 5. Conclusions

This study presents a folate-targeting and GSH-responsive PEG shielding antibody prodrug strategy, and based on this strategy, designed and synthesized two prodrugs: FA-PEG-S-Atz (a PD-L1-blocking prodrug) and FA-PEG-S-Hu5 (a CD47-blocking prodrug). By integrating “folate-mediated active targeting” and “TME-dependent PEG cleavage,” this strategy not only enhances the tumor specificity of immune checkpoint inhibitors (ICIs) but also effectively reduces their off-target toxicity. It is expected to provide a safer and more precise prodrug technology for immune checkpoint therapy.

## Data Availability

The original contributions presented in this study are included in the article/Supplementary Materials. Further inquiries can be directed to the corresponding authors.

## Supplementary Materials

The following supporting information can be downloaded at: https://www.mdpi.com/article/10.3390/molecules30214292/s1, Figure S1: The 1H NMR of PEG10k-NHS; Figure S2: The 1H NMR of folate-PEG10k-NHS; Table S1: Materials.
