# Supplementary material for: Glutathione-Responsive Folate-Targeted Prodrugs: Tumor-Specific PD-L1 and CD47 Blockade
Source: Molecules. 2025 Nov 5;30(21):4292. doi: 10.3390/molecules30214292 (PMC12608855; doi:10.3390/molecules30214292)
Supplement: Supplementary file 1 [file molecules-30-04292-s001.zip › molecules-3912655-supplementary.pdf]

Supplementary Information for

Glutathione-Responsive Folate-Targeted Prodrugs: Tumor-Specific PD-L1 and CD47 Blockade

Jianfeng Wang, Lianqi Liu, Dian Xiao *, Fei Xie * and Xinbo Zhou *

This file includes:

Supplementary Figure S1. The ^{1}H NMR of PEG10k-NHS.

Supplementary Figure S2. The ^{1}H NMR of folate-PEG10k-NHS.

Supplementary Table S1. Materials.

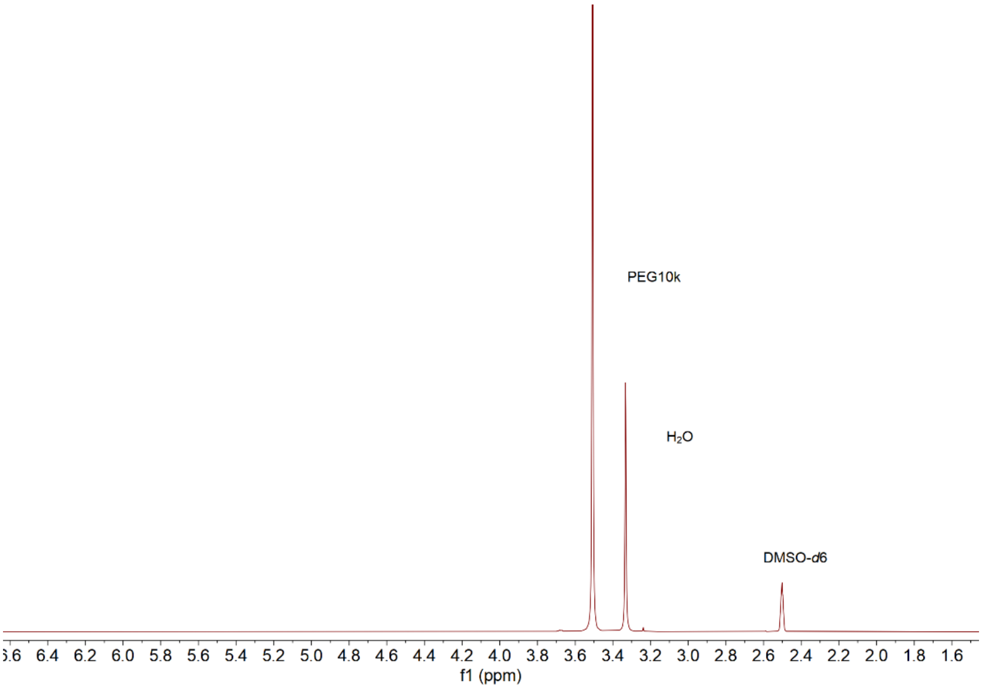

Supplementary Figure S1. The ^{1}H NMR of PEG10k-NHS.

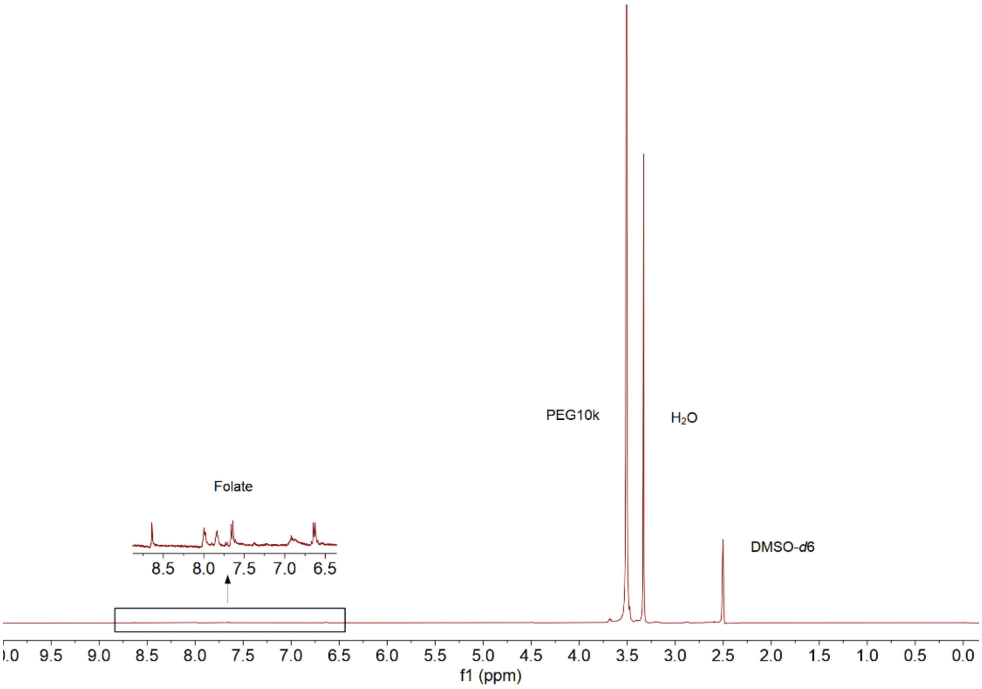

Supplementary Figure S2. The ^{1}H NMR of folate-PEG10k-NHS.

Supplementary Table S1. Materials.

Name	Source	Usage, Dilution
Atezolizumab	Roche<br>MA, Germany	Functional
Hu5f9	MedChemExpress (HY-P99029)<br>Shanghai, China	Functional
FA-PEG5k/10k-S-NHS	RUIXIBIO.Ctd (customization)<br>Xi'an, China	Functional
PEG5k/10k-S-NHS	RUIXIBIO.Ctd (customization)<br>Xi'an, China	Functional
PD-L1 Recombinant Protein	SinoBiological (10084-H08H)<br>Beijing, China	SPR, 25 μg/mL;<br>ELISA, 1 μg/mL
CD47 Recombinant Protein	SinoBiological (12283-H08H)<br>Beijing, China	SPR, 25 μg/mL;<br>ELISA, 1 μg/mL
DiD membrane Fluorescent Probe	Beytime (C1995S)<br>Shanghai, China	Colocalization live-cell imaging, 10 μM
